# The efficacy of bidirectional barbed sutures for incision closure in total knee replacement: A protocol of randomized controlled trial

**DOI:** 10.1097/MD.0000000000021867

**Published:** 2020-08-21

**Authors:** Zijian Ye, Wengang Zhu, Xinhua Xi, Qiang Wu

**Affiliations:** Department of Orthopedics, Yuebei People's Hospital, Shaoguan, Guangdong, China.

**Keywords:** bidirectional barbed sutures, complication, protocol, total knee replacement

## Abstract

**Background:**

Barbed suture is a novel type of suture introduced in different surgical specialties. Nevertheless, its effect in total knee replacement is still unclear in terms of wound complications and cost effectiveness. The purpose of the present work is to evaluate the safety and efficacy of bidirectional barbed suture in reducing postoperative wound complications in the patients undergoing total knee replacement.

**Methods:**

This prospective, randomized, and controlled study was performed from January 2017 to December 2018. It was authorized via institutional review committee of Yuebei People's Hospital (GDYB1002189). Hundred participants were divided randomly into 2 groups, namely, control group (n = 50) and the study group (n = 50), respectively. All operations were performed using the Miller-Galante prosthesis (Zimmer; Warsaw, IN). For study groups, the joint capsule (Stratafix1-0) and subcutaneous (Stratafix2-0) and intracutaneous (Stratafix3-0) tissues were sutured by a bidirectional barbed suture. At the end, extra 4 to 5 stitches were made to avoid detachment and incision rupture. For control group: the joint capsule was sutured by a traditional absorbable suture (Ethicon VICRYL∗ Plus 1-0), and the subcutaneous tissue was sutured by an absorbable suture (Ethicon VICRYL∗ Plus 2-0). The skin was sutured by staples. Incision length, suture time, operation time, postoperative length of hospital stay, and incision complications (such as effusion, infection, hematoma, and skin necrosis) were recorded. All data analyses are implemented through utilizing SPSS for Windows Version 20.0.

**Results:**

The results will be shown in Table 1.

**Conclusion:**

This study can reach a reliable evidence for utilizing bidirectional barbed suture in wound closure in total knee replacement.

**Trial registration:**

This study protocol was registered in Research Registry (researchregistry5823).

## 1 Introduction

Total knee replacement (TKR) is a common surgical treatment for patients with end-stage osteoarthritis.^[1,2]^ The number of TKR has sharply increased with the development of technology and concept. It is reported that more than 300 thousand primary TKR procedures are performed annually in the United States.^[3,4]^ Therefore, improving cost-effectiveness will be beneficial. The efficiency of TKR can be improved from a variety of aspects, such as reducing the postoperative complications, rapid rehabilitation, improving the surgical techniques, and the managements of pain and blood products.^[5–7]^ Prevention of complications, particularly infections, will be of the utmost importance, due to the treatment of an infectious TKR will cost at least 4 times as many resources as treating the original TKR.^[8,9]^

Meticulous wound closure is very important for a successful TKR. However, its importance is usually overlooked. Inadequate suturing methods may cause wound-healing problems that could increase the risk of infection. The surgical duration is also a hazard factor for the postoperative wound infection, which increases the risk by 8% per minute.^[10]^ Barbed suture is a novel type of the suture introduced in different surgical specialties since 2009. The suture system was authorized by the United States Food and Drug Administration and by Health Canada for soft tissue in 2004, and it has been on the market in the United States since 2007.^[11]^ Previous studies have reported that using the barbed sutures to close incision in a variety of surgeries was related to a reduction of wound complications and operation time in various surgeries.^[12,13]^ Despite all potential advantages, barbed suture is not commonly used in TKR. This might be due to its higher cost and uncertain clinical benefits. Previous articles on barbed sutures in TKR is limited and has yielded conflicting results. Campbell^[14]^ reported that the incidence of complications associated with wound closure in the patients undergoing TKR were obviously higher in wounds closed with the barbed locking sutures than with the staplers, while others observed that they decrease cost and closure time.

The purpose of the present work is to evaluate the safety and efficacy of bidirectional barbed suture in reducing postoperative wound complication in the patients undergoing TKR. We hypothesized that bidirectional barbed suture can improve the efficiency of TKR without increasing the risk of wound complications.

## 2 Materials and methods

### 2.1 Study design

This prospective, randomized, and controlled study was performed from January 2017 to December 2018 which was performed in accordance with the SPIRIT Checklist for randomized studies. It was authorized via the Institutional Review Committee in Yuebei People's Hospital (GDYB1002189) and then was registered in research registry (researchregistry5823). Each patient received the written informed consent. The subjects in this research were 100 primary TKR patients from Yuebei People's Hospital. The following are the inclusion criteria:

(1) the patients with end-stage knee osteoarthritis requiring primary TKR;
(2) the patients that were over 60 years old and they could cooperate with our treatment and the postoperative observation.

The exclusion criteria included the following:

(1) a history of coagulopathy, a history of deep vein thrombosis or pulmonary embolism 3 months before the operation;
(2) patients with neurovascular diseases of the affected limbs;
(3) renal failure or placement of an arterial stent within the past year.

### 2.2 Randomization and blinding

Hundred participants were divided randomly into 2 groups, namely, control group (n = 50) and the study group (n = 50), respectively. A table of random numbers hidden in the 1:1 ratio was computer-formed. A researcher who did not take part in the trial used the website Randomization.com to generate a random distribution sequence, which was hidden in sealed opaque sequence numbered envelopes that were allocated to investigators. The surgeons, investigator, anesthetist, and nurses were all kept blinded to allocation results.

### 2.3 Surgical procedure

All patients were given the general anesthesia. The surgical procedures were performed by the senior surgeon. In the process of operation, pneumatic tourniquet was utilized. An incision was made in the center of the knee and then extended to the medial side of patella. All TKRs were performed using the Miller-Galante prosthesis (Zimmer; Warsaw, IN). For study groups, the joint capsule (Stratafix1-0) and subcutaneous (Stratafix2-0) and intracutaneous (Stratafix3-0) tissues were sutured by a bidirectional barbed suture. At the end, extra 4 to 5 stitches were made to avoid detachment and incision rupture. For control group: the joint capsule was sutured by a traditional absorbable suture (Ethicon VICRYL∗ Plus 1-0), and the subcutaneous tissue was sutured by an absorbable suture (Ethicon VICRYL∗ Plus 2-0). The skin was sutured by staples. The intra-articular tranexamic acid (1 g in the normal saline [10 mL]) is a routine hemostatic drug in our institute. All the patients in this study were followed up in outpatient for 2 weeks, 2 months, and 3 months.

A gait rehabilitation program was conducted by a physiotherapist, and the patient began walking with a walker on the first day after surgery. Mechanical thromboprophylaxis combined with the chemoprophylaxis was utilized for the prevention of the venous thromboembolism. As a kind of chemical thromboprophylaxis, all the patients were subcutaneously injected with a low heparin molecular weight (ie, 0.2 mL and 2000 IU) 6 hours after the surgery, and a full dose of (4000 IU, 0.4 mL) was used at 24 hours intervals after the surgery.

### 2.4 Data collection

Pre-operative and postoperative clinical data were evaluated by an independent senior surgeon blinded to the patient's randomization. Incision length, suture time, operation time, postoperative length of hospital stay, and incision complications (such as effusion, infection, hematoma, and skin necrosis) were recorded. Western Ontario and McMaster Universities Arthritis Index were collected preoperatively and 2 weeks, 2 months, and 3 months in outpatient clinic. Length of stay also were recorded in detail.

### 2.5 Statistical analysis

All data analyses are implemented through utilizing SPSS for Windows Version 20.0. All the data are represented with proper characteristics as median, mean, percentage as well as standard deviation. Mann–Whitney *U* test or the independent samples *t*-test were used to analyze the inter group comparison. Chi-square detection was utilized to compare the categorical variables among the groups. A *P* < .05 was regarded the significant in statistics.

## 3 Results

The results will be shown in Table 1.

**Table 1:**
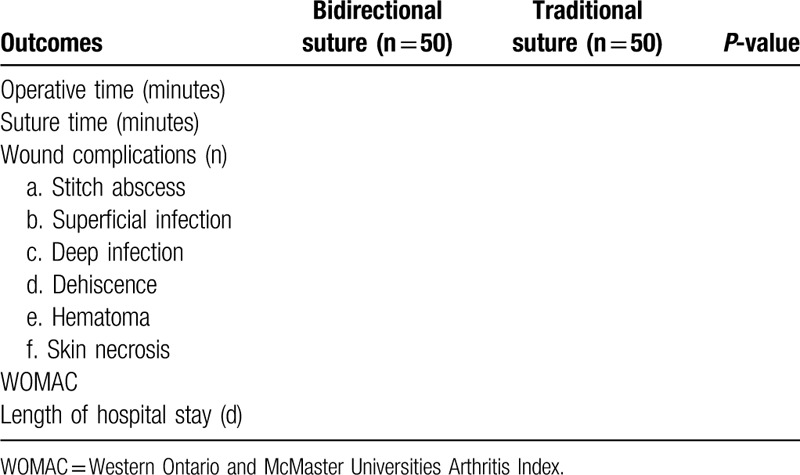
Study results.

## 4 Discussion

In the past few years, a new class of sutures – barbed suture has been introduced by the industry.^[15]^ Barbed sutures eschew the traditional, smooth, knot-requiring characteristic of sutures in favor of barbs that anchor the suture to the tissues without a knot. The barbed sutures have 3D tiny barbs that are uniformly distributed on the surface. A novel suturing device with a self-anchorage system can maintain tissue tension and requires no knotting after the sutures are strained.^[16]^

TKR wound is traditionally sutured by intermittent knots using common sutures. The absorption of large knots may lead to severe local tissue inflammations and potential infectious lesions. It is reported that the highest rates of reoperation after TKR for the treatment of postoperative wound problems were related to wound infection (21%), wound hematoma (13.9%), and wound necrosis (14.3%). In uncomplicated cases, infection resulted in reoperation in 0.82%.^[17]^ Galat^[18]^ reported that wound patients requiring early surgical treatment for wound-healing problems after primary TKR are at significantly increased risk for further complications, including deep infection and/or major subsequent surgery, specifically, resection arthroplasty, or muscle flap coverage. Thus, effective and safe closure of incisions is critical to increase surgical efficiency and prevent infections in patients. We further conduct a double-blinded randomized controlled trial to assess the efficacy and safety of the bidirectional barbed suture in reducing postoperative wound complication in the patients undergoing TKR. The strength of this study was its prospective and randomized design.

## 5 Conclusion

This study can reach a reliable evidence for utilizing bidirectional barbed suture in wound closure in TKR.

## Author contributions

**Data curation:** Wengang Zhu.

**Investigation:** Xinhua Xi.

**Writing – original draft:** Zijian Ye.
